# Interrelationships between sleep quality, circadian phase and rapid eye movement sleep: Deriving chronotype from sleep architecture

**DOI:** 10.3758/s13428-025-02671-w

**Published:** 2025-04-21

**Authors:** Csenge G. Horváth, Bence Schneider, Borbála Rozner, Míra Koczur, Róbert Bódizs

**Affiliations:** 1 Institute of Behavioural Sciences, Semmelweis University, Nagyvárad Tér 4. 20 Floor, 1089 Budapest, Hungary; 2 Institute for Biology, University of Veterinary Medicine, Budapest, Hungary

**Keywords:** REM phase, Core body temperature, Actigraphy, Chronotype, Sleep quality

## Abstract

The relationship between sleep quality, circadian rhythms, and REM sleep has not been deliberately investigated in previous scientific reports. Here, we aim to examine the associations between these factors by specifically focusing on the temporal dynamics of REM sleep in all night records, as well as to provide a new, objective, EEG-derived chronotype indicator. To achieve those aims, a wearable EEG headband recorded home sleep database was analyzed in terms of total sleep time (TST), REM dynamics, core body temperature, wrist actigraphy, Munich Chronotype Questionnaire, Pittsburgh Sleep Quality Index, subjective morning sleep quality, and Likert Sleepiness Scale. Furthermore, records from the Budapest-Munich database of polysomnography (PSG) were analyzed for REM sleep patterns, TST, arousal dynamics, and age. The results show that the timing of the crest of REM propensity (REM_maxprop_) reliably correlated with weekly average actigraphy sleep midpoints, subjective chronotype measures, and also tended to be associated with core body temperature. Additionally, REM_maxprop_ emerged at earlier times in children and middle-aged participants as compared to teenagers and young adults. Subjective sleep quality exclusively reflected the shortening of headband-recorded sleep as compared to weekly average TST. REM percent negatively correlated with NREM arousal density. It can be concluded that the overnight REM sleep dynamic (REM_maxprop_) is a putative indicator of circadian phase/chronotype with potential relevance for home sleep studies. However, sleep quality indices are less conclusive in between-subjects design, urging the need for longitudinal investigations allowing interindividual analyses.

## Introduction

Efficient, satisfactory sleep is a complex concept that is difficult to characterize along a single dimension or using only one type of metric. Besides focusing on objective PSG measures, such as duration, continuity, or composition, the proper timing of sleep driven by the circadian component of sleep regulation is also an important factor. Optimal timing relies on the internally generated circadian rhythm showing individual-specific synchronization to, and phase alignment with, rhythmic environmental ques (Earth’s rotation around its own axis and consequent day–night alternation). The interindividual differences in the phase of entrainment of the internal and environmental rhythms are treated within the context of the chronotype concept. The latter is frequently assessed by means of questionnaires targeting habitual sleep–wake schedules (Roenneberg et al., 2022). Although there are several available objective methods for determining circadian phase non-invasively such as mobile core body temperature measurement or actigraphy recordings, the estimation of chronotype is not a usual part of routine diagnostic or research-related examinations of sleep. This represents a significant gap in the field not only because participants with different chronotypes are in different circadian phase during the same period of sleep recording (Bódizs et al., 2022) but also because of the known association of chronotype with both health-related and behavioral factors such as heart rate variability (Sűdy et al., 2019), cardio-vascular disease (Bhar et al., 2022), cognitive performance, age-related changes or different personality traits, etc. (Montaruli et al., 2021; Roenneberg et al., 2004). This incompleteness may be due to the complex, time- and/or money-consuming protocols and the subjective nature of questionnaires applied in the field. In a recent paper, we have already suggested a non-rapid eye movement (NREM) EEG-derived chronotype indicator, which is the nadir of sleep spindle frequency during the sleep period (Horváth & Bódizs, 2024b). Although this serves as a promising indicator of the circadian phase, detecting and analyzing the frequency of sleep spindles are not usually necessary to answer the original research or diagnostic question in routine sleep examination settings. Thus, a metric that can be derived directly from sleep architecture could help bridge the above-mentioned gap. In terms of sleep medicine and research, the main benefit of such a metric is that it eliminates the need for additional devices, multi-day assessments, or resource-intensive protocols beyond the usual PSG recording. Moreover, it offers greater objectivity compared to measurements influenced by individual choices, such as bedtimes or habitual sleep–wake schedules. Furthermore, the utility in determining chronotype from previously collected PSG data could afford a major advantage in retrospective studies.

Nap studies and continuous bedrest experiments suggest that REM sleep is governed by circadian regulation processes. Its distribution throughout a 24-h day is mostly systematic with the highest REM amount in the early morning (Endo et al., 1981). Furthermore, REM propensity has a temporal relationship with the circadian rhythm of the core body temperature (see review in Wurts & Edgar, 2000).

Besides its sensitivity to circadian modulation, the amount of REM sleep was found to be associated with subjective sleep quality in the next morning (Della Monica et al., 2018; Pierson-Bartel & Ujma, 2024). Due to the circadian timing of REM sleep propensity and the relationship between sleep quality and REM sleep duration, REM sleep was proposed as a sleep quality indicator in a literature review (Barbato, 2021). Furthermore, in a recent study, the duration of REM sleep in daytime sleep was found to interact with the adaptation to night shift work, where the group with the larger circadian adaptation had longer REM sleep duration (Zimberg et al., 2024).

As REM amount and REM propensity are the most frequently reported variables related to circadian modulation, we aim to propose a circadian phase indicator that includes both of the above-mentioned aspects of REM sleep. This variable, called the crest of REM propensity (marked hereon as REM_maxprop_), is the middle time of the sleep cycle when the ratio of REM sleep is the highest during the sleep period. It encompasses both propensity and duration of REM sleep, as it describes the time when NREM is the shortest relative to the subsequent REM sleep period. Secondly, the accumulation of REM sleep duration prior to the REM_maxprop_ is also analyzed, as this variable (hereafter referred to as REM_acc_) is hypothesized to provide insight into both the circadian phase and the association between sleep quality and REM duration described in the literature mentioned above. However, as sleep efficiency/continuity and total sleep time (TST) are the most consistently reported correlates of subjective sleep quality and subsequent sleepiness during the day (see, e.g., in Åkerstedt et al.,  2013, 2016; Della Monica et al., 2018; Pierson-Bartel & Ujma, 2024) their interrelationship with REM sleep variables is also an important question.

Overall, the present study aims to examine the relationship between REM sleep and circadian phase, objective sleep quality as reflected in sleep continuity measured by the number and normalized density of arousals, overall subjective and morning sleep quality, as well as morning sleepiness.

The main assumption of the present study is the convergent validity of REM_maxprop_ as a chronotype indicator. Thus, we hypothesize that it will positively correlate with subjective chronotype, actigraphy-derived sleep midpoint, and with the time of CBT minimum of the participants (meaning later time of REM_maxprop_ for participants with later subjective chronotype, later time of actigraphy sleep midpoint and later CBT minimum). Furthermore, we hypothesize that both REM_maxprop_ and REM_acc_ will reflect age-related differences in the circadian rhythm and sleep quality, respectively.

Secondly, we suggest that REM-derived metrics, mainly REM_acc_ and REM percent, will be associated with subjective and objective measures of sleep quality as proposed by the literature discussed above.

## Methods

### Devices and databases

Analyses were conducted on two databases (Table 1), one with whole-night polysomnographic- (Budapest-Munich database, recorded between 2003 and 2006) and another with whole-night wearable headband EEG recordings derived from different studies initiated in 2020. The former database was used in order to provide wide age range and a high number of subjects involved in the analyses.

**Table 1:** Sample sizes, study protocols, and measurements used in the different datasets

				Sample			Measurements				Questionnaires
Database	Dataset	Study protocol	Reference	*N*	Female	Age range (years)	Method	No. of recording days/nights	Analyzed nights (based on availability)	Device	
Budapest-Munich			(Bódizs et al., 2022)	251	122	4–69	Polysomnography	2	2nd night	Ambulatory PSG	-
Wearable EEG headband	Study 1	Sleep deprivation	(Horváth & Bódizs, 2024a)	45	24	18–39	Electroencephalography	2	Baseline sleep	Hypnodyne Zmax EEG headband	PSQI, MCTQ, Likert Sleepiness Scale
							Actigraphy	7	First 5 nights ending with awakenings from baseline sleep (min. 4 nights)	Geneactive Original wristband accelerometer	
	Study 2	Regular sleep schedule with actigraphy and CBT	-	15	9	20–42	Electroencephalography	1	All	Hypnodyne Zmax EEG headband	PSQI, MCTQ, Likert Sleepiness Scale (1–10), morning sleep quality (very bad to very good)
							Actigraphy	7	All (min. 6)	Geneactive Original wristband accelerometer	
							Non-invasive core body temperature meas	48 h	All	Calera research heat-flux sensor	
		Regular sleep schedule	-	8	5	18–45	Electroencephalography	1	All	Hypnodyne Zmax EEG headband	
	Study 3	General life circumstances	-	22	13	19–59	Electroencephalography	3	1 (the one with best signal quality)	Hypnodyne Zmax EEG headband	PSQI, MCTQ, Likert Sleepiness Scale (1–10), morning sleep quality (very bad to very good)
							Actigraphy	7	6	Geneactive Original wristband accelerometer	
							Non-invasive core body temperature meas	Min. 72 h	All	Calera research heat-flux sensor	

The Budapest-Munich database consists of 251 (122 females) subjects’ signals recorded in different laboratories (electrodes were placed according to the standard 10–20 system) with participants in the age range of 4–69 years divided into four age groups: children (*N* = 31, 4–10 years), teenagers (*N* = 36, 11–20 years), young adults (*N* = 150, 21–40 years), and middle-aged adults (*N* = 34, 41–69). A detailed description of the database with information on sampling rates, precision, electrode- and recording locations can be found in Bódizs et al. (2022). In the present study, only EMG, ECG, and EEG C3 channels (for the purpose of automatic arousal detection), along with the manually scored hypnograms of the sleep recordings, were used.

The headband EEG database comprised 90 adults’ data from three different studies (age range 18–59, mean age 25 years). In these studies, all participants slept in their own homes; thus, sleep was recorded in an ecologically valid environment with a Hypnodyne Corp. Zmax EEG headband. The recording device has a sampling rate of 256 Hz at derivations F7-Fpz and F8-Fpz. The three studies included in our current analyses followed different protocols, but each was designed to include at least one night of habitual sleep recorded with a mobile headband, after which participants rated their sleepiness on a 1–10 Likert scale. Additionally, participants completed questionnaires on overall sleep patterns, including the Munich Chronotype Questionnaire (MCTQ) and the Pittsburgh Sleep Quality Index (PSQI), as well as a 5 to 7-day actigraphy recording using the Geneactiv Original wrist-worn accelerometer. Recording devices were the same in all three studies.

The first study involved 45 (24 females) healthy young adults (age range 18–39 years) participating in a 7-day-long examination with a 35-h-long sleep deprivation protocol in the last 2 days. The first 5 days were free of specific instructions and interventions; only actigraphy wearing was required, and baseline sleep of the sleep deprivation protocol was recorded on the fifth night of the week using the EEG headband. This sleep period was self-scheduled and ad libitum with freely chosen bedtimes in the evening, with prohibited alarm clock usage in the morning (more detailed protocol can be found in G. Horváth & Bódizs, n.d.). In the second study, healthy adult participants (*N* = 23, 14 females) had to follow a regular sleep schedule adapted to their general/habitual routine for 1 week, during which the completion of sleep diaries and sleepiness questionnaires were required before and after the sleep periods. On the sixth evening, participants recorded their sleep with the headband. A subset of these participants (*N* = 15, nine females) also wore a non-invasive core body temperature (CBT) sensor (CALERA research; sampling rate: 1 Hz) in the first 48 h and a wristband accelerometer (Geneactive Original) during the whole week. Finally, the third study is a larger ongoing research project focusing, among others, on the usefulness of wearable devices in the research of circadian rhythm. According to the study protocol, participants are examined with mobile wearable devices in their everyday life circumstances. In this week-long study, a 7-day wrist actigraphy and a Cortrium C3 + Holter ECG measurement were conducted; furthermore, 3-day-long CBT and EEG measurements were conducted along with morning and evening sleep diaries. From this study, *N* = 22 participants’ CBT, actigraphy, one night of EEG data, as well as morning sleep quality and sleepiness ratings after the EEG-recorded sleep episode, were included in the present analyses. To sum up the EEG headband database, habitual home sleep records (baseline sleep in the sleep deprivation study), the first 5–7 days of actigraphy measurements (first five nights in the sleep deprivation study ended with awakening from baseline sleep, 6–7 nights for other studies depending on availability), CBT data, sleepiness ratings and sleep quality ratings from sleep diaries from the mornings of the EEG-recorded sleep, MCTQ, and PSQI results were analyzed.

All subjects were free of psychiatric or neurological disorders based on self-reports. In addition, the first two datasets in the wearable EEG database have the same exclusion criteria, including the Hungarian version of the Pittsburgh Sleep Quality Index (Takács et al., 2016) score over 5, Beck Depression Inventory (Beck et al., 1961) score over 12 (moderate and severe depression symptoms; Rózsa et al., 2001), extreme circadian preference (MCTQ chronotype scores outside of the ± 3 SD of reported values in young Hungarian subjects according to Haraszti et al. (2014) and shift work, as well as reported acute and/or chronic medical diagnoses or ongoing pharmacological treatments. The third wearable device study aims to focus on the general population. As a consequence, we only excluded subjects with acute health issues from this subsample of the current report. However, only one participant was (10 min) further than 3 SD from the population mean as indicated by MCTQ results (sleep midpoint range for the three datasets: 01:40:00 AM to 7:35:00 AM, mean 4:19:35 AM) thus, we excluded this participant from the statistical analyses.

The National Public Health Centre Institutional Committee of Science and Research Ethics or the Ethics Committee of the Semmelweis University (Budapest, Hungary), as well as the Medical Faculty of the Ludwig Maximilians University (Munich, Germany), approved the research protocols, and the whole experiment was implemented in accordance with the Declaration of Helsinki. Every participant (or, in the case of minors, their parents or guardians) provided informed consent to participate in the study.

### Analyses of the electroencephalography data

**Fig. 1:**
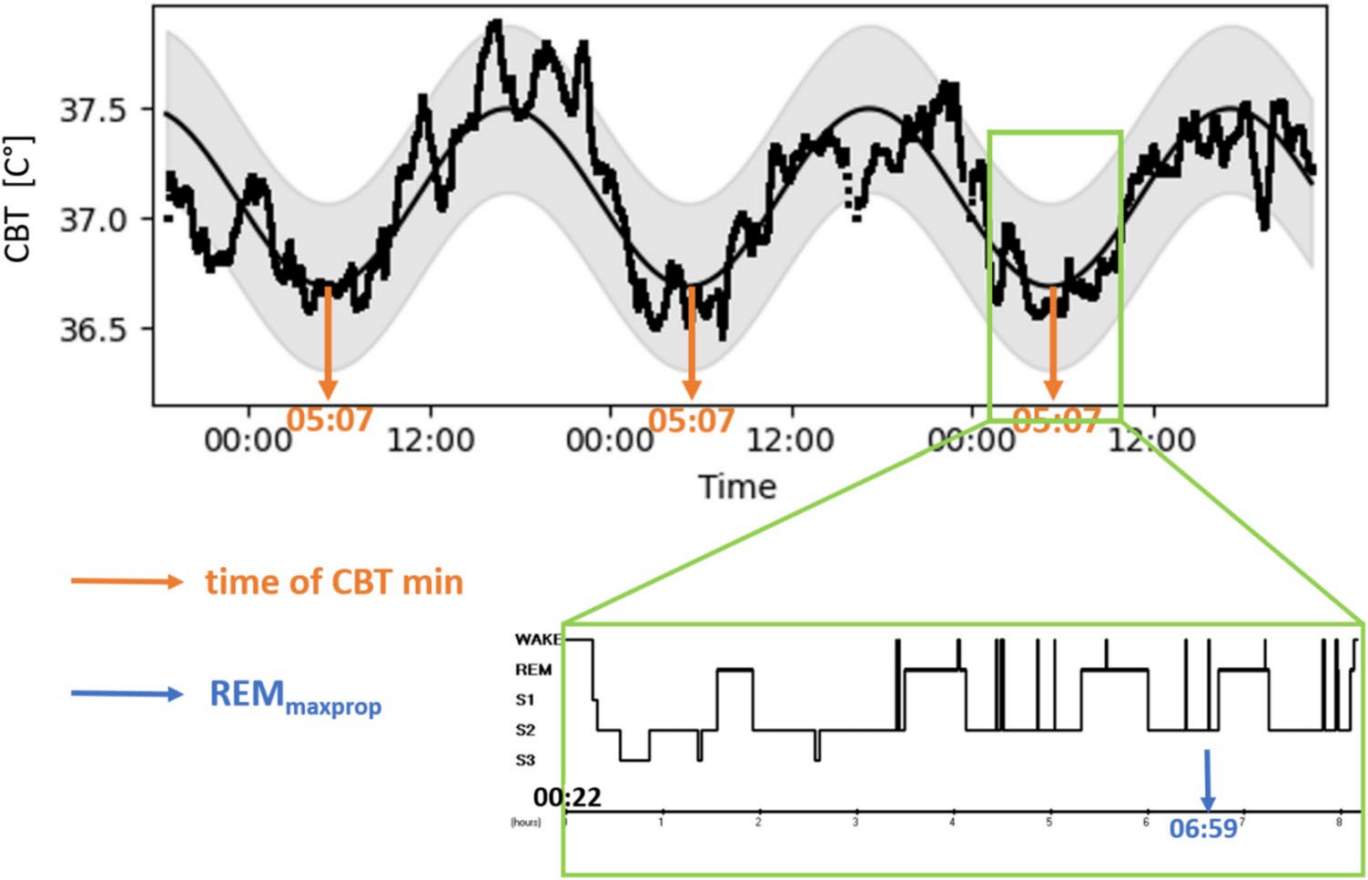
REM_maxprop_ and CBT minimum time of a 19-year-old female participant. *Note. Top*: CBT data and the fitted sinusoid via cosinor analysis, with the time of minimum indicated by the *orange arrows*. Since the curve fitting was applied to the entire recording, the local minima of the cosinor curve are identical across all three nights. *Bottom*: hypnogram of the same subject, where the *blue arrow* indicates the estimated time of REM_maxprop_ (i.e., sleep cycle midpoint when the ratio of REM sleep is the highest compared to the preceding NREM period)

All PSG recordings were scored in 20-s epochs manually according to the standard criteria of sleep–wake states (Berry et al., 2018). For the headband dataset, we followed the same scoring rules as for PSG data; however, in the Zmax recordings, some physiological channels are replaced with other measurements with nearly the same information content compared to the originally recommended derivations. Instead of ECG, photoplethysmography (PPG) is applied, while chin EMG is replaced by a triaxial accelerometer (indicating head movements), although in the latter case, muscle artifacts are also spectacular in the signal. Due to the frontal EEG derivations, REM sleep-related eye movements are also clearly detectable in the waveform. Additional support for scoring sleep in Zmax recordings is provided by the skin temperature signal, an acoustic noise channel (which measures the intensity of external noise), and a light sensor (which provides information about the wearer’s environment). The resulting hypnograms were split into sleep cycles (successive NREM and REM episodes; Fig. 1). The analyzed polygraphic variables included total sleep time (TST), REM percent (total REM duration relative to total sleep duration), local time of the largest increase in REM/NREM ratio during the course of the night (REM_maxprop_*), and accumulated REM sleep duration prior to the time of REM_maxprop_. Where polysomnography (PSG) data were recorded along with C3, EMG and ECG channels (Budapest-Munich database), the total number of arousals, as well as the number of arousals separately in REM and NREM sleep were detected with an automatic algorithm developed by Fernández-Varela and colleagues (Fernández-Varela et al., 2017) and validated on different heterogeneous databases (Alvarez-Estevez & Fernández-Varela, 2019). The density of arousals was also calculated for the total and separately for REM and NREM periods, defined as the arousal count/sleep duration (/REM/NREM duration).

*REM_maxprop_ is the putative circadian phase indicator of REM sleep proposed in the present study (see example in Fig. 1). Its calculation was as follows:

1. Calculate the duration of REM and NREM periods for every sleep cycle separately.
2. Estimate the ratio of the REM/NREM periods in each sleep cycle by dividing REM duration by NREM duration.
3. Look for the sleep cycle that contains the highest REM/NREM ratio.
4. Determine the middle of the sleep cycle containing the highest REM/NREM ratio and estimate the local time of this cycle-middle from the start time of the sleep recording.

### Subjective sleep quality and circadian phase indicators

Actigraphy-derived weekly average sleep midpoint, time of the CBT minimum, and subjectively measured chronotype were used as validated circadian phase indicators (Reid, 2019; Roenneberg et al., 2019; Santisteban et al., 2018).

Actigraphy sleep midpoints were calculated from sleep onset and wake-up times for all days separately, then the whole week (5–7 days) average of the sleep midpoints was used in the subsequent analyses. Sleep periods, thus sleep onset and wake-up times were estimated with GGIR, an open-source R package (Migueles et al., 2019; Van Hees et al., 2015), using the setting of time window as wake-to-wake.

The CBT signal was subjected to cosinor analysis with the use of an open-source algorithm, CosinorPy (Moškon, 2020). Finally, the time of the first local minimum of the fitted sinusoid on the time series was used as the circadian phase indicator (example in Fig. 1).

Subjective chronotype was derived from the Munich Chronotype Questionnaire (MCTQ), which considers the oversleeping adjusted sleep midpoint (MSFsc: estimated from self-reported bed- and wake times on work- and free days) as a circadian phase indicator (Roenneberg et al., 2003).

Subjective sleep quality was measured in two ways. The PSQI score was used as a measure of general subjective sleep quality. In addition, self-ratings of sleep quality (ranging from very bad to very good on a 1–5 scale) and sleepiness (on a 1–10 Likert scale) were collected in the morning following the night sleep period assessed by the EEG headband.

### Statistical analyses

Statistical analyses were conducted with TIBCO Statistica Software. An a priori power analysis was conducted for the determination of the sufficient size of the sample to test our hypotheses. Its results revealed that the required sample size to achieve 80% power for detecting a medium effect (according to Cohen’s guidelines) at a significance level of α = 0.05, was *N* = 68 for correlational analysis and *N* = 79 for one-way ANOVA. Thus, the planned sample size of *N* = 90 (wearable EEG headband database) and *N* = 251 (BPM database) appeared suitable to test the study hypotheses regarding the associations between EEG and subjective variables.

The two databases were analyzed separately using the following statistical tests. Associations among variables were examined with Pearson correlation (coefficients marked with r in the result section) where the data have Gaussian distribution and Spearman’s rho (marked with R in the result section) was estimated when the data is non-normally distributed, or ordinal (PSQI score, Likert sleepiness, morning sleep quality). Group comparisons were conducted with one-way ANOVA on normally distributed data and with Kruskal–Wallis ANOVA where a non-parametric version of the test was needed.

## Results

### REM sleep indices, chronotype, and subjective sleep quality (wearable EEG headband dataset)

Out of the 90 recordings, 80, 77, and 35 subjects have complete, analyzable EEG, actigraphy, and CBT recordings, respectively. Regarding the questionnaires, MCTQ MSFsc cannot be calculated for six individuals as they reported the use of an alarm clock on weekends, and a further one had to be excluded as the MSFsc of this participant was 10 min above the 3 SD distance from the population mean. Additionally, two participants forgot to fill out the sleepiness scale after waking up from the EEG-recorded night sleep; however, all subjects who had to fill out a morning sleep diary gave reported successfully on their subjective sleep quality after the EEG-night sleep (from here on in the text referred to as morning sleep quality). Detailed sample sizes for the different analyses can be seen in Table 2.

**Table 2:** Sample sizes of the different analyses

	EEG	Actigraphy	CBT	MCTQ	Sleepiness scale	Current sleep quality	PSQI
EEG	80						
Actigraphy	67	76					
CBT	31	34	34				
MCTQ	73	71	30	83			
Sleepiness scale	77	74	32	81	87		
Current sleep quality	41	36	34	37	42	44	88
PSQI	78	76	34	82	86	44	89

Number of participants with coexisting measures

**Fig. 2:**
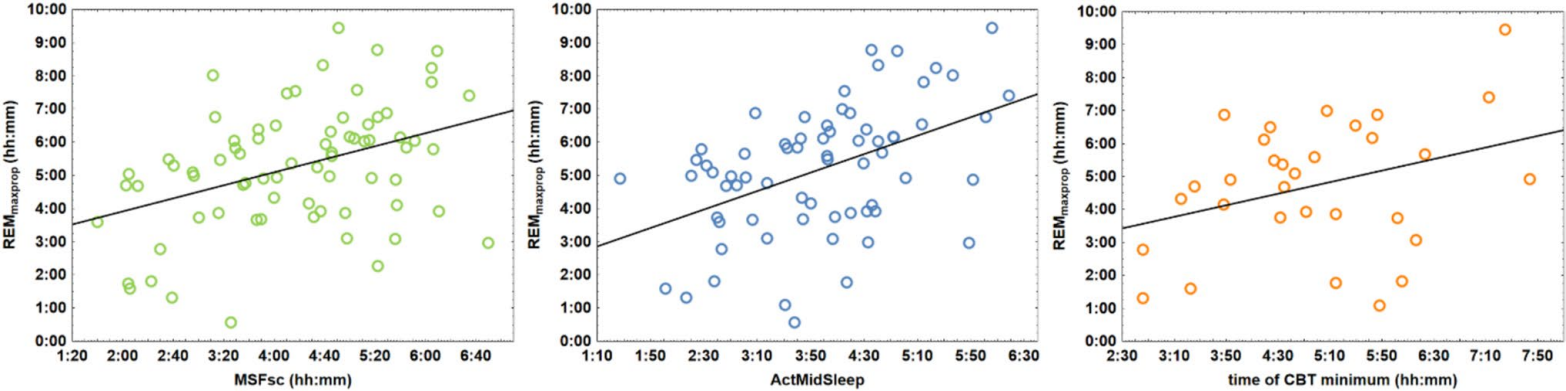
Associations of REM_maxprop_ with the different circadian phase metrics. *Note.* Correlation of REM_maxprop_ with MSFsc, weekly average sleep midpoint regarding actigraphy, and with the time of the CBT minimum

Validated chronotype and circadian phase indicators significantly correlated with the proposed putative EEG-derived circadian phase marker defined herein as REM_maxprop_, except CBT, which was characterized by a tendency level association (MCTQ: *r* = 0.4, *p* < 0.0001; Act_MidSleep_: *r* = 0.47, *p* < 0.0001, CBT: *r* = 0.34, *p* = 0.06, Fig. 2). In contrast REM_acc_ did not correlate with formerly established circadian phase markers (MCTQ: *r* = –0.013, *p* = 0.9, Act_MidSleep_: *r* = – 0.04, *p* = 0.74, CBT: *r* = – 0.09, *p* = 0.65). In addition, all validated chronotype indicators correlated significantly with each other (MCTQ vs. Act_MidSleep_: *r* = 0.7, *p* < 0.0001; MCTQ vs. CBT: *r* = 0.53, *p* = 0.003; Act_MidSleep_ vs. CBT: *r* = 0.62, *p* < 0.0001).

Subjective sleep quality indicators were not significantly associated with REM-related variables or EEG TST (Table 3).

**Table 3:** Spearman’s rho-s and *p* values of correlations between subjective sleep quality measures and EEG variables

	PSQI		Morning sleep quality		Sleepiness	
	R	p	R	p	R	p
REM_maxprop_	– 0.09	0.43	– 0.1	0.52	0.01	0.96
REM_acc_	– 0.19	0.09	0.14	0.4	– 0.2	0.09
REM percent	– 0.15	0.18	0.04	0.8	– 0.1	0.37
EEG TST	– 0.18	0.11	– 0.03	0.86	– 0.15	0.20

Since the weekly average total sleep time was significantly longer than TST on the EEG-measured night (m_ActTSTavr_ = 7:57 hh:mm, SD = 00:55 hh:mm; m_TST_ = 7:35, SD = 1:09 hh:mm; *t*(67) = 3.53, *p* < 0.001) we examined whether this difference in sleep duration between the average and EEG-night was reflected in the subjective quality of sleep assessed in the morning of the EEG-night sleep. We found a tendency for shorter sleep time to be associated with lower sleep quality. Specifically, participants who had shorter EEG-night TST compared to their average sleep duration (greater difference) reported lower sleep quality ratings upon awakening (TST_avr_–TST_EEGnight_ & current sleep quality: *r* = – 0.33, *p* = 0.06); however, this was not true for the sleepiness ratings (TST_avr_–TST_EEGnight_ *r* = 0.15, *p* = 0.22. We also checked whether REM percent is associated with the difference of TST_avr_ & TST_EEGnight_, as early morning REM loss is highlighted in the literature as a possible cause of sleep quality reduction (Barbato, 2021; Naiman, 2017). We found no significant association between the two variables (REM percent vs. TST_avr_–TST_EEGnight_: *r* = – 0.21, *p* = 0.089).

### REM sleep, age, and objective sleep quality (Budapest-Munich database)

The arousal detection was completed for *N* = 232 subjects’ recordings, as 19 participants did not have a full-night EMG channel necessary for reliable detection. Other analyses regarding age and REM timing or duration were performed on the whole sample (*N* = 251). The sample sizes of the age groups were different: children: *N* = 31, teenagers: *N* = 36, young adults: *N* = 131, middle-aged adults: *N* = 34. A Levene’s test of homogeneity of variances was performed to test the inhomogeneous variances between the different age groups. Age groups were characterized by homogeneous variances regarding REM_maxprop_ (*F*(3,244) = 1.55, *p* = 0.2)_,_ but not regarding REM_acc_ (*F*(3,247) = 5.7, *p* < 0.01), thus in this latter case the result of Welch’s F-test is reported which corrects for variance heterogeneity.

**Fig. 3:**
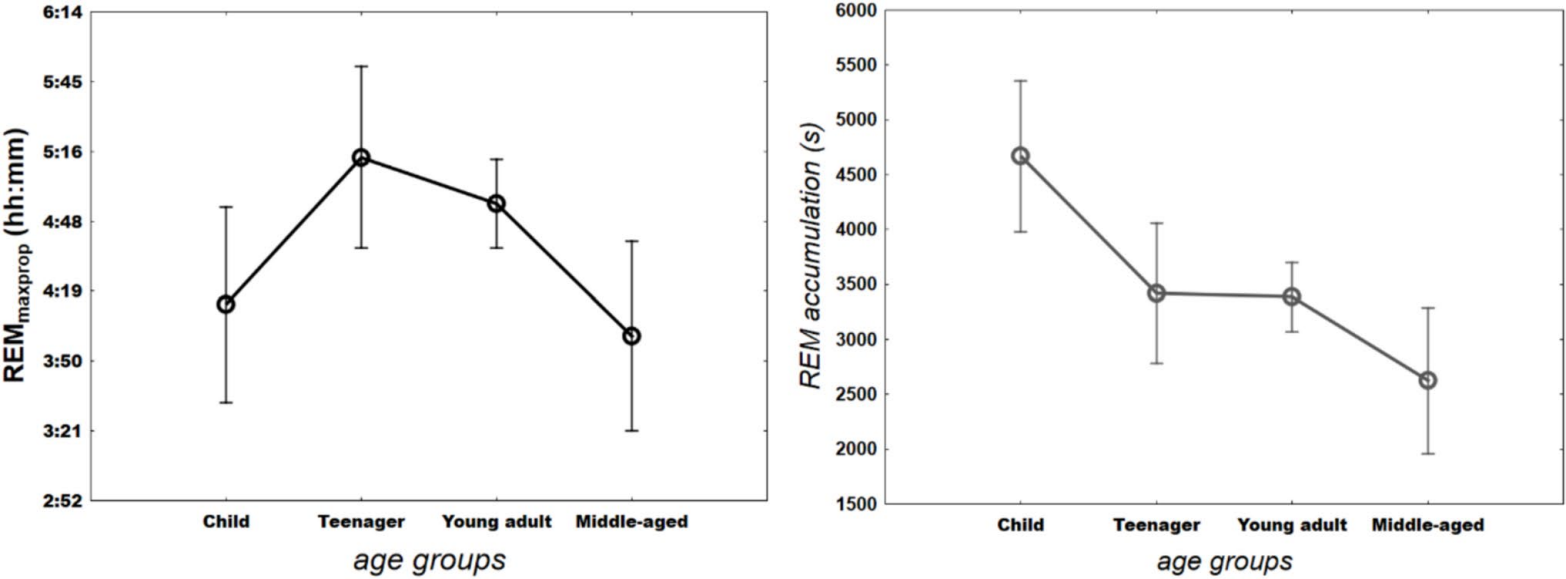
The timing of REM_maxprop_ and accumulation of REM sleep prior to the maximal propensity as a function of age

Results of one-way ANOVA showed that age has an effect on both REM_maxprop_ and REM accumulation (Fig. 3). REM_maxprop_ seems to occur earlier in children and middle-aged groups as compared to teenagers and young adults (*F*(3,244) = 3.66, *p* = 0.01, partial η^2^ = 0.04). Furthermore, age-related changes in REM accumulation prior to REM_maxprop_ are reflected in the differences between age groups regarding REM_acc_ (Welch’s *F*(3) = 4.8, *p* = 0.004).

Objective sleep quality indicators (total-, REM-, and NREM- arousal count) proved to correlate significantly and positively with EEG-defined TST, indicating higher number of arousals revealed in longer sleep periods. Total arousal count, however, is not associated with REM-related variables. REM percent negatively associated with NREM arousal count and was characterized by a positive correlation with REM arousal count. Finally, while REM_acc_ is only associated with the REM arousal count, REM_maxprop_ does not have any association with any arousal count metric (Table 4).

**Table 4:** Pearson correlation coefficients and the corresponding *p* values for the associations between arousal- and EEG-related metrics

Arousals	Total count		Count in NREM		Count in REM		Total density		Density in NREM		Density in REM	
	*r*	*p*	*r*	*p*	*r*	*p*	*r*	*p*	*r*	*p*	*r*	*p*
REM_maxprop_	0.06	0.39	– 0.04	0.58	0.07	0.31	– 0.01	0.896	– 0.1	0.224	– 0.06	0.408
REM_acc_	– 0.01	0.843	– 0.03	0.62	**0.26**	< 0.001*	**– 0.14**	0.036*	– 0.11	0.086	– 0.04	0.569
REM percent	– 0.12	0.065	**– 0.13**	0.049*	**0.42**	< 0.001*	**– 0.21**	0.001*	**– 0.18**	0.006*	– 0.04	0.596
EEG TST	**0.2**	0.003*	**0.15**	0.025*	**0.29**	< 0.001*	– 0.1	0.159	– 0.04	0.53	– 0.07	0.301

Significant correlations are marked with an asterisk

After normalizing the number of arousals with the length of sleep (arousal count/hour in different sleep phases and during the whole sleep period), correlational analyses revealed no association between TST and any of the arousal density metrics. However, significant negative associations of total and NREM sleep arousal densities with REM percent were revealed. Likewise, higher total arousal density predicted lower REM_acc_ (Table 4). Furthermore, Kruskal–Wallis ANOVA revealed a significant effect of age groups on all three arousal density metrics (H(3)_total_ = 32.4, *p* < 0.001, η^2^_H_ = 0.12; H(3)_NREM_ = 29, *p* < 0.001, η^2^_H_ = 0.11; H(3)_REM_ = 20.5, *p* < 0.001, η^2^_H_ = 0.07).

## Discussion

The present study examines the relationship of REM sleep with the circadian phase as well as with objective and subjective sleep quality. We found that the time of the maxima in REM/NREM ratio (REM_maxprop_) reliably reflects the individual differences in the circadian phase and chronotype, the latter measured with both subjective and objective metrics. Statistically significant correlations were found between the proposed circadian phase marker (REM_maxprop_) and previously published (questionnaire- and actigraphy-derived) measures, whereas the level of the association between REM_maxprop_ and CBT_min_ emerged at a trend level. However, the accumulation of REM prior to REM_maxprop_ (REM_acc_) was not associated with measures of the circadian phase, although age seemed to affect this variable (decrease of REM_acc_ with increasing age). Finally, no direct proof of the role of REM-related variables in subjective and objective sleep quality was revealed. Nevertheless, less sleep relative to the weekly average sleep duration tended to be associated with lower subsequent self-reported sleep quality which indicates a within-subject association between the objective and subjective metrics, cohering with earlier reports (Åkerstedt et al., 2013; Pierson-Bartel & Ujma, 2024), as well as a first-night type of effect which could indeed be operative in home sleep studies conducted with self-applicable electrode sets (Miettinen et al., 2018).

Early studies show that REM sleep is promoted by the circadian pacemaker as the crest of its rhythm is preceded by the minimum of the CBT rhythm (Dijk & Czeisler, 1995). However, an easy-to-measure phase indicator was never proposed. REM_maxprop_ is a promising indicator of circadian timing as it correlates with self-reported chronotype and actigraphy-derived sleep midpoint. Furthermore, the earlier REM_maxprop_ of children and middle-aged adults as compared to teenagers and young adults must be mentioned. It is well known that chronotype and circadian timing shift to later phases during the transition from childhood to adolescence, whereas this phase delay is followed by a gradual phase-advance with aging (Duffy et al., 2015; Roenneberg et al., 2004). These age effects in the circadian phase align with our results regarding the dependence of REM_maxprop_ on the age of the subjects. Although REM_acc_ was also affected by age, it seemed to better reflect the age-related changes in REM percent which is reducing during the life span (Floyd et al., 2007; Lokhandwala & Spencer, 2022). Indeed, our results did not support the circadian modulation of this variable, as it was not associated with any of the circadian phase indicators analyzed here.

The present study does not equivocally support the role of REM sleep as an indicator of sleep quality, as no association was found between EEG variables and the different subjective indicators of sleep- and wake quality. However, if we consider arousal count and density as an objective indicator of sleep quality in accordance with several reports in the literature (Zakevicius et al., 2013), REM percent seems to provide information on the integrity of the sleep process, as it correlates negatively with the abundance of NREM arousals. Our findings indirectly suggest that frequent interruptions of NREM sleep by arousals might hinder the emergence of REM sleep. Accordingly, reduced REM duration was found to emerge as one of the most common sleep architectural change across different health problems (Ujma & Bódizs, 2024). Although there were moderate associations between REM_acc_, REM percent, and the count of arousal in REM sleep, these relationships are confounded by the duration of sleep time as longer TST was also correlated with more arousal events. In spite of the fact that none of the subjective indicators were reflected in the EEG-metrics, a promising picture emerged when the difference of weekly and current sleep duration was compared to subjective sleep quality. The relationship between subjective and objective sleep quality has always been a contradictory question. However, longitudinal studies found within-subject association between subjective sleep quality and the preceding sleep period (Åkerstedt et al., 2013). In a recent study, robust interindividual differences and stable intraindividual dynamics of sleep cycles and sleep stage transitions were reported (Kishi & Van Dongen, 2023). It is likely that the interindividual differences in sleep architecture and sleep duration overshadow the relationship between objective and subjective metrics. Thus, we think that examining “poor sleep” in healthy people requires more than one-night-long sleep recordings performed in ecologically valid settings, which could form the basis for a within-participant analysis of the recordings and self-ratings.

The limitations of the study include the inability to perform circadian analyses and assess sleep continuity within the same sample, and the lack of data on extreme chronotypes, which prevents extrapolation of our findings to those groups. Another limitation is the use of EEG headbands instead of the gold-standard PSG for examining circadian phase. However, this choice also serves as an advantage, given the ecologically valid environment in which the studies were conducted. In sum, our study proposes a new, putative REM-derived EEG indicator of the circadian phase. It encourages further research on the association between subjective and objective sleep quality in longitudinal settings but found no evidence supporting the relevance of circadian regulation – sensitive aspects of REM sleep among the proposed sleep quality indicators. Although the all-night REM percentage partially reflects arousal density, it does not correspond to subjective sleep quality or actual sleep amount relative to the weekly average. Further studies are needed to unravel the relationship between REM sleep and sleep quality.

## Data Availability

The datasets generated during and/or analyzed during the current study are available in the OSF repository: https://osf.io/4acjp/. None of the reported studies were preregistered.
